# Interstitial expansion in pressure overload left ventricular hypertrophy

**DOI:** 10.1186/1532-429X-15-S1-O92

**Published:** 2013-01-30

**Authors:** Thomas A Treibel, Steven K White, Daniel Sado, Filip Zemrak, Sanjay M Banypersad, Andrew Flett, Mark Caulfield, Anna S Herrey, Steffen E Petersen, James Moon

**Affiliations:** 1 The Heart Hospital Imaging Centre, University College London, London, UK; 2 The Hatter Cardiovascular Institute, University College London Hospitals NHS Trust, London, UK; 3 Cardiovascular Biomedical Research Unit, Barts and the London School of Medicine and Dentistry, Queen Mary University of London, London, UK

## Background

Diffuse myocardial fibrosis (DMF) is an important factor in cardiac disease, but until recently could only be accurately assessed with biopsy. We hypothesised that DMF measured by Equilibrium contrast CMR (EQ-CMR) is elevated in cardiac pressure overload (hypertension and aortic stenosis), that the degree of DMF will track the clinical severity of pressure overload and cardiac effects, and as such DMF may be a key biomarker in assessing the cardiac effects of pressure overload.

## Methods

ECV measurement was by EQ-CMR. The T1 mapping sequence was ShMOLLI. The contrast agent was Gadoterate meglumine (Dotarem) at 0.1mmol/Kg (bolus) plus infusion at 15 minutes at 0.0011 mmol/kg/min. CMR was at 1.5T (Siemens Avanto):

ECV = (1-hematocrit)x (1/T1)myo ÷ (1/T1)blood.

ECV was measured in 43 patients with isolated, well-controlled hypertension (median age 56, range 21 to 78, 55% male), 28 patients with severe aortic stenosis (AS) undergoing aortic valve replacement (median age 70, range 60 to 84, 71% male), and 50 healthy volunteers (median age 47, range 28 to 69, 58% male).

## Results

**Figure 1:**
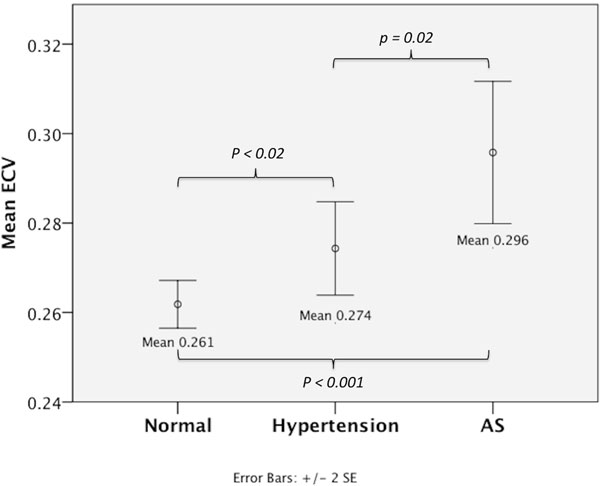
ECV measurements by EQ-CMR were the lowest in the control group with significantly higher ECV values in hypertension and aortic stenosis (0.261 versus 0.274 versus 0.296,p≤0.02).

ECV measurements were the lowest in the control group with significantly higher ECV values in hypertension and AS (0.261 versus 0.274 versus 0.296, p≤0.02; Figure 1).

**Figure 2:**
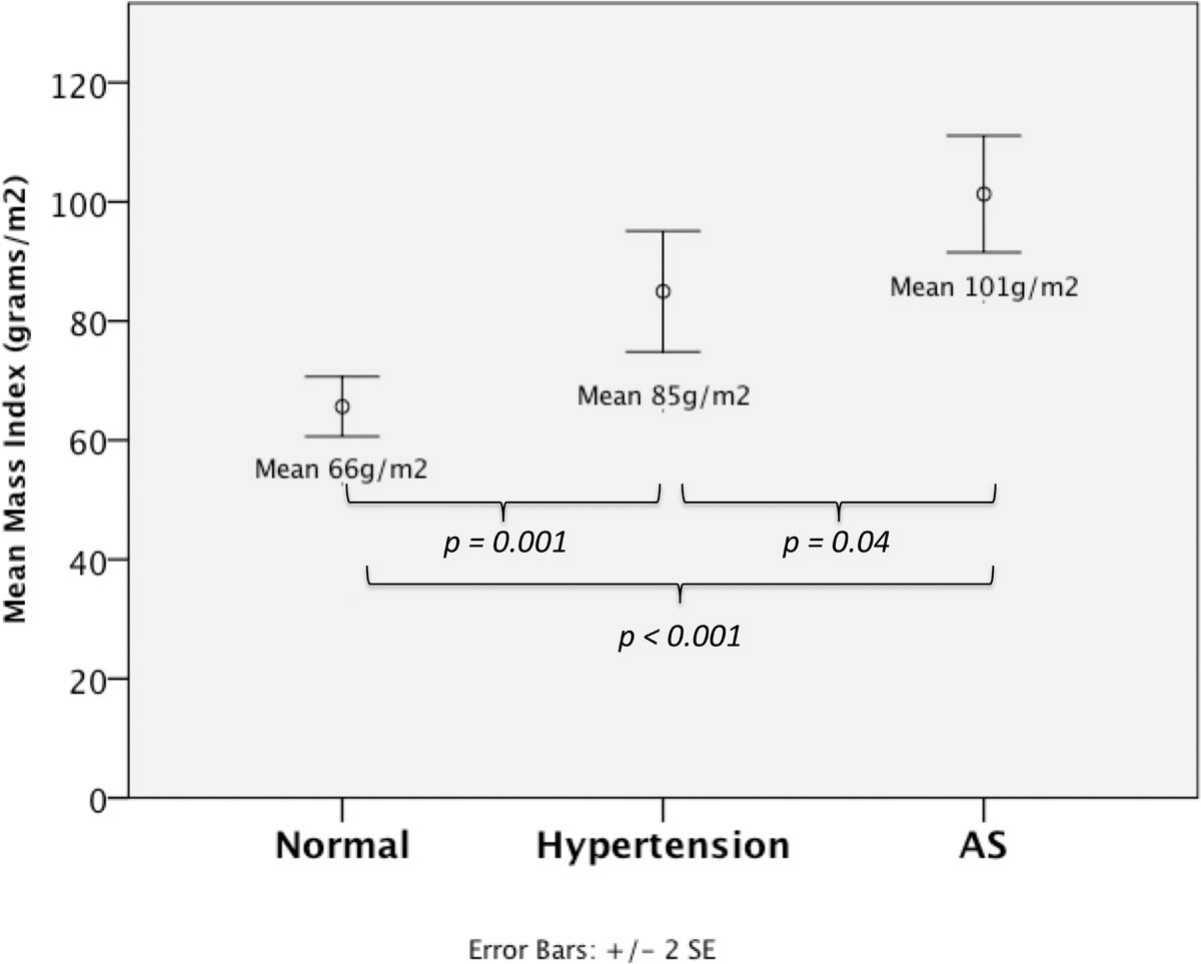
The left ventricular mass index increased from normal to hypertension to aortic stenosis (66 g/m^2^ versus 85 g/m^2^ versus 101 g/m^2^, p<0.05).

The mass index increased from normal to hypertension to AS (66 g/m^2^ versus 85 g/m^2^ versus 101 g/m^2^, p<0.05; Figure 2), whereas the indexed left atrial area was only significantly higher in AS (11.0 and 11.6 cm^2^/m^2^ versus 13.2 cm^2^/m^2^, p<0.05); end-systolic and diastolic volumes were not significantly different among cohorts.

## Conclusions

The myocardial ECV increases with the degree of left ventricular pressure overload hypertrophy.

## Funding

British Heart Foundation; National Institute for Health Research.

